# Editorial: Mixed Infections of Plant Viruses in Nature and the Impact on Agriculture

**DOI:** 10.3389/fmicb.2022.922607

**Published:** 2022-06-07

**Authors:** Yi Xu, Murad Ghanim, Yong Liu

**Affiliations:** ^1^Department of Plant Pathology, Nanjing Agricultural University, Nanjing, China; ^2^Key Laboratory of Soybean Disease and Pest Control (Ministry of Agriculture and Rural Affairs), Nanjing Agricultural University, Nanjing, China; ^3^Department of Entomology, Volcani Center, Rishon LeZion, Israel; ^4^Hunan Plant Protection Institute, Hunan Academy of Agricultural Sciences, Changsha, China

**Keywords:** plant virus, mixed infections, synergism, antagonism, insect vector

Plants are grown under adverse conditions of multiple plant viruses in the field, and inevitably, mixed infections are common. Several important virus diseases of plants are the outcomes of interactions between different viral agents. An example is maize lethal necrosis, a devastating disease caused by the synergistic combination of corn chlorotic mottle virus and potato Y virus (Wangai et al., 2012; Redinbaugh and Stewart, 2018). Although significant advances have been made toward understanding the biology of individual viruses, in most cases, the diverse outcomes resulting from within-host interactions between viruses in mixed infections are poorly understood.

In nature, mixed infections with two or more plant viruses are frequent in plants, interacting in multiple and intricate ways. The objectives of this Research Topic were to provide a platform for researchers interested in plant viruses to share their recent results related to the various aspects of mixed infections: synergistic, antagonistic and neutral interactions, virus–plant host/vector interactions, ecology and control strategies. A total of 4 research articles have been contributed by 29 authors to the topic with more than 4,600 views to date.

High-throughput sequencing (HTS) technologies have become indispensable tools to characterize plant virus diversity, being very powerful to characterize mixed infections as virtually all viruses present in a plant sample might be identified without prior sequence knowledge (Adams et al., 2009; Al Rwahnih et al., 2009; Donaire et al., 2009; Kreuze et al., 2009; Villamor et al., 2019). In this Research Topic, Miljanić et al. used HTS to identify the viruses and viroids in preclonal candidates of grapevine (*Vitis vinifera* L.) varieties from a Slovenian wine-growing region. A complete description of coinfection in each plant was well documented. Interestingly, they showed that no grapevine was found to be virus- and viroid-free, and no grapevine was found to be infected with only one virus or viroid, while the highest number of viral entities in a plant was eight, indicating that mixed infections of viruses and viroids is the rule. These results will improve the early diagnostics of preclonal candidates of grapevine varieties, which is especially important to control virus diseases in grapevines at their early growing stage.

In nature, most of the plant viruses are transmitted horizontally by insect vectors, and deciphering virus–vector interactions are an emerging research subject in plant virology (Whitfield et al., 2015; Dietzgen et al., 2016). Two research articles published in this Research Topic deal with vector transmissions which are affected by mixed infections of different viruses. Chen et al. experimentally analyzed the interactions between the four causal agents of the tobacco bushy top disease (TBTD) in symptom induction and aphid transmission by successful construction of infectious clones. They showed clear evidence that only co-inoculation of these four causal agents to tobacco plants could cause typical TBTD symptoms. Moreover, they showed that the successful transmission of tobacco bushy top virus (TBTV, an umbravirus), TBTV satellite RNA, and tobacco vein distorting virus (TVDV) satellite RNA by *Myzus persicae* depended on the presence of TVDV (a polerovirus), while the presence of TBTV satellite RNA increased the aphid transmission efficiency of TBTV and TVDV, indicating a mutual interaction between different viruses and their associated satellites. Jia et al. studied another interesting field within the scope of this Research Topic. In addition to the synergistic effect of rice stripe mosaic virus (RSMV), a newly discovered plant cytorhabdovirus, and rice gall dwarf virus (RGDV) on viral replication potential and pathogenicity in rice plants, these authors also found that RGDV significantly promoted the propagation of RSMV when co-infecting the vector, *Recilia dorsalis*. Accordingly, co-infection significantly promoted the acquisition and transmission efficiencies of RSMV by *R. dorsalis*, Showing that the synergy between plant viruses also takes place in the transmitting vectors.

A mixed virus infection that results in more severe symptoms is usually referred to synergistic interactions (Syller, 2012). Tatineni et al. analyzed the results of infection in wheat with up to four viruses in different combinations. The outcome of the infections was evaluated by observations of symptoms as well as measurement of the amount of viral RNA and coat protein. Interestingly, for some virus combinations, stronger virus symptoms were not accompanied by increasing virus titers. This study shows that there are complex antagonistic and synergistic interactions between viruses in field-grown crops. Overall, the papers in this Research Topic reveal different perspectives of current research on mixed infections of plant viruses in nature, from staple crop studies to investigations into the important cash crops and then into the intricate synergistic effects on the tripartite interactions between viruses, plants, and vectors. Illuminating the mechanisms of these mixed infections is crucial for understanding viral pathogenesis and evolution and, consequently, developing efficient and stable control strategies in the field.

## Author Contributions

YX and YL wrote the first draft of the editorial. MG revised the draft and added additional sections. All authors contributed to the article and approved the submitted version.

## Funding

This work was partially supported with funding provided by the National Natural Science Foundation of China Grant 32172376, the Startup Fund for Distinguished Scholars from Nanjing Agricultural University to YX.

## Conflict of Interest

The authors declare that the research was conducted in the absence of any commercial or financial relationships that could be construed as a potential conflict of interest.

## Publisher's Note

All claims expressed in this article are solely those of the authors and do not necessarily represent those of their affiliated organizations, or those of the publisher, the editors and the reviewers. Any product that may be evaluated in this article, or claim that may be made by its manufacturer, is not guaranteed or endorsed by the publisher.
